# Pott’s Puffy Tumor in Young Age: A Systematic Review and Our Experience

**DOI:** 10.3390/jcm13216428

**Published:** 2024-10-26

**Authors:** Antonio Daloiso, Tiziana Mondello, Francesco Boaria, Enrico Savietto, Giacomo Spinato, Diego Cazzador, Enzo Emanuelli

**Affiliations:** 1 Otolaryngology Section, Department of Neuroscience DNS, University of Padova, 35100 Padova, Italy; antoniodaloiso96@gmail.com (A.D.); tiziana.mondello1995@gmail.com (T.M.);; 2 Otorhinolaryngology and Skull Base Center, AP-HP, Hospital Lariboisière, 75010 Paris, France; 3 Otolaringology Unit, Ca’ Foncello Hospital, Local Health Unit N.2 “Marca Trevigiana”, 31100 Treviso, Italy

**Keywords:** acute sinusitis, frontal sinusitis, Pott’s puffy tumor, intracranial complication, pediatric sinusitis, acute sinus infection, orbital complications of sinusitis, bacterial sinusitis, frontal bone osteomyelitis, subperiosteal abscess

## Abstract

**Background**: Pott’s Puffy Tumor (PPT) in young-age patients is a rare clinical entity characterized by osteomyelitis of the frontal bone with a subperiosteal abscess collection. Previous reviews primarily consist of small, retrospective case series and anecdotal reports. This study aims to present the largest, most up-to-date systematic review of essential clinical findings, diagnostic modalities, microbiologic considerations, and treatment approaches for managing PPT in pediatric and adolescent populations. **Methods**: PubMed, Scopus, and Web of Science databases were systematically screened until 3 January 2024. The protocol of this investigation was registered on PROSPERO in January 2024, and the systematic review was performed according to the PRISMA statement. The study included 184 patients from 109 articles and an additional case from the authors’ institution. **Results**: PPT commonly stems from untreated rhinosinusitis, respectively, acute pansinusitis, frontal acute rhinosinusitis and chronic rhinosinusitis, and direct head trauma. Infections typically involve a polymicrobial anaerobe-predominant microbiome. Computed tomography and magnetic resonance imaging are routinely used for presurgical assessment and posttreatment surveillance. Intracranial complications were significantly associated with the type of surgical treatment (*p* value < 0.0001). **Conclusions:** PPT is a significant and relatively morbid disease often under-recognized and misdiagnosed due to its variable clinical presentation. Management includes both antimicrobial therapy and surgical intervention, emphasizing the importance of an interdisciplinary approach.

## 1. Introduction

Pott’s Puffy Tumor (PPT) is defined as one or more subperiosteal abscesses of the frontal bone associated with underlying osteitis and osteomyelitis [1]. It was first described by Sir Percivall Pott, who related it to earlier forehead trauma in 1768 and to earlier frontal sinusitis in 1775 [2]. It appears as localized swelling of the forehead, with inflammatory signs, tenderness, and swelling of the overlying skin. Associated typical symptoms are headache, periorbital swelling, rhinorrhea, fever, vomiting, and lethargy [1]. PPT is often an indicator of intracranial complications [1]. The infection has the potential to extend into the intracranial cavity through bony erosions, pre-existing pathways, or septic thrombosis via the Haversian canals. This propagation can lead to severe intracranial complications such as meningitis, epidural abscess, subdural empyema, intracerebral abscess, and dural sinus thrombophlebitis [1]. In such cases, computed tomography (CT) is employed for treatment planning, while magnetic resonance imaging (MRI) plays a crucial role in the detection of intracranial complications [2].

PPT was once considered a rare occurrence in the post-antibiotics era, with the majority of reported cases involving adolescents and young adults. This increased susceptibility in the younger age group can be attributed to two main factors: the heightened vascularity of their diploic system and the relative increase in blood supply to the still-developing frontal sinuses [3]. Despite advancements in early detection, targeted antibiotic treatments, and surgical interventions, the morbidity and mortality associated with intracranial complications of sinusitis have significantly decreased. However, intracranial complications still manifest, necessitating swift diagnosis and multidisciplinary treatment to prevent long-term neurological sequelae and fatalities [4]. This study aims to present an up-to-date systematic review of the etiological, clinical, surgical, as well as microbiological findings related to PPT in young patients. Additionally, it aims to foster discussion on the treatment course, which remains a topic of controversy in the literature.

## 2. Materials and Methods

### 2.1. Protocol Registration

The protocol of this systematic review was registered on PROSPERO, an international database of prospectively registered systematic reviews in health and social care (Center for Reviews and Dissemination, University of York, York, UK), in January 2024 (registry number CRD42024498741).

### 2.2. Search Strategy

A systematic literature review was conducted according to the Preferred Reporting Items for Systematic Reviews and Meta-Analyses (PRISMA) recommendations [5]. The electronic databases Scopus, Pubmed, and Web of Science were searched from database inception to 3 January 2024. A combination of MeSH terms (“sinusitis” [MeSH Terms], “intracranial complication” [MeSH Terms]) and free-text words (“Pott’s puffy tum*”, “acute sinusitis”, “intracranial complication”) were utilized to search. The reference lists of all the included articles were thoroughly screened to find other relevant articles. References were exported to Zotero bibliography manager (v6.0.10, Center for History and New Media, George Mason University, Fairfax, VA, USA). After duplicates removal, two reviewers (A.D. and T.M.) independently screened all titles and abstracts and then evaluated the full texts of the eligible articles based on the inclusion criteria. Any disagreement between the reviewers involved in the literature search was resolved through discussion with all authors to reach a consensus.

### 2.3. Selection Criteria

Studies were deemed eligible when the following inclusion criteria were met: (i) confirmed diagnosis of PTT; (ii) patients ≤ 18 years old. Exclusion criteria were as follows: (i) lack of relevant data; (ii) non-original studies (i.e., reviews, recommendations, editorials, conference papers, clinical challenges, and book chapters); (iii) animal model studies; (iv) non-English studies.

### 2.4. Data Extraction and Quality Assessment

Extracted data were collected in an electronic database including first author, year of publication, sample size, number of patients included, age of the patients, gender, etiology, imaging tests used, types of complication, types of surgery, medical treatment and culture, and outcome. The quality of the studies eligible for inclusion was categorized as Poor, Fair, and Good, in agreement with the National Institute of Health’s quality assessment tool for Observational Cohorts and Cross-Sectional Studies (https://www.nhlbi.nih.gov/health-topics/study-quality-assessment-tools, accessed on 3 January 2024) [6]. Two reviewers (A.D. and T.M.) independently evaluated the papers, and any disagreement was resolved by discussion.

### 2.5. Statistical Analysis

Qualitative and quantitative analyses of the data (variance of each variable and descriptive statistics measures of central slope and variability) were performed. Cases were distinguished as intracranial complication or no intracranial complication. Using Chi-square, a statistical association analysis was run between the aforementioned cohorts and the categorical demographic, intraoperative, and postoperative variables. Post hoc analysis for multiple comparisons of categorical data was applied with Bonferroni’s correction. This test assessed each hypothesis at a significance level of α/n, where α is the overall significance level and n is the number of hypotheses being tested. Two-tailed *p* < 0.05 was considered statistically significant. SPSS version 20 for Windows (IBM Corp, Armonk, NY, USA) was used for all statistical analyses.

## 3. Results

### 3.1. Case Report

**Figure 1:**
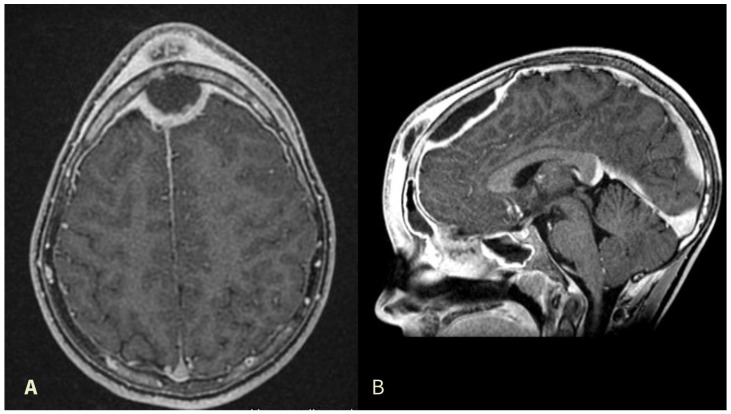
Preoperative contrast-enhanced MRI image revealing subcutaneous forehead swelling and a frontal epidural abscess in axial (**A**) and sagittal (**B**) views.

A 12-year-old, Italian male with no comorbidities complained of nasal congestion and headache for one month, who was initially treated by his general practitioner with oral amoxicillin (1 g every 12 h for a week). He presented thereafter to the emergency department clinic with progressively increasing frontal swelling and eyelid edema. He underwent cerebral and head and neck contrast-enhanced CT scan and MRI that revealed a complicated frontal sinusitis with bone erosion of the posterior wall of the frontal sinus and an epidural collection with peripheral rim enhancement at the frontal lobe, suggestive of an epidural abscess with compression of sagittal sinus (Figure 1). He was transferred to our hospital where he received intravenous (IV) vancomycin 40 mg/kg/day, metronidazole 20 mg/kg/day, and ceftazidime 2 g/day. Ophthalmological evaluation excluded orbital complications. 

**Figure 2:**
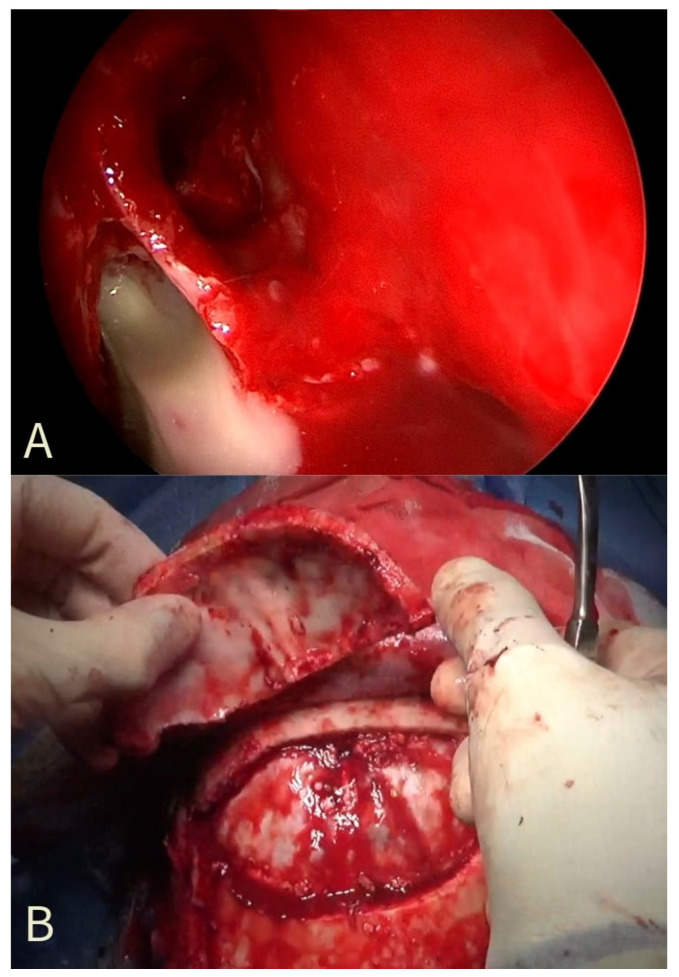
Intraoperative photographs. (**A**): an endoscopic view of the discharge of pus from the frontal sinus; (**B**): the Pott Puffy Tumor during subperiosteal dissection with inner plate erosions from the epidural granulation tissue.

The patient underwent Endoscopic Sinus Surgery (ESS) with a right total ethmoidectomy, right frontal sinusotomy (Draf IIa) (Figure 2A), and maxillary antrostomy. Concomitant craniotomy with trephination and drainage of the brain abscess was performed for the epidural empyema and subperiosteal abscess (Figure 2B). The craniotomy operculum was rebuilt with a custom titanium plate because of the osteomyelitis which involved the frontal bone. Microbiological analysis of the purulent material demonstrated growth of *Streptococcus intermedius*. The patient remained hospitalized in our department for 6 weeks, and he received IV antibiotics throughout the entire hospital stay. The follow-up MRI performed 6 weeks after surgery revealed the resolution of the subperiosteal and epidural abscesses. He was discharged with saline irrigative 3 times/day with no clinical symptoms and no radiological signs. He continued oral antibiotics for another 4 weeks. Informed consent was obtained for publication purposes.

### 3.2. Search Results and Quality Assessment

**Figure 3:**
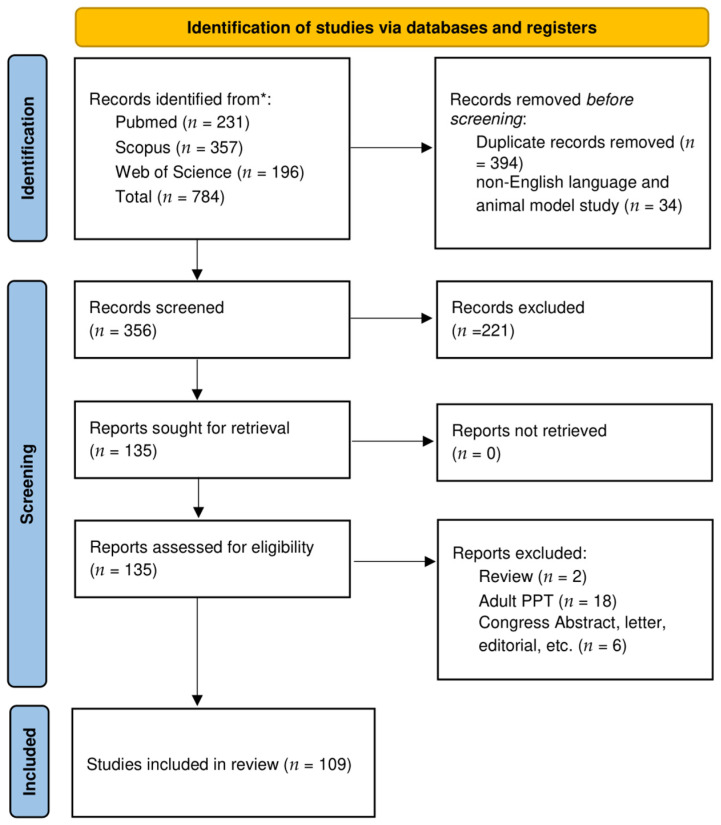
PRISMA diagram representing the Electronic Database Search and inclusion/exclusion process of the review. Legend * date of last search: 3 January 2024.

After duplicates removal and exclusion of 221 records due to coherence with the inclusion/exclusion criteria, 135 articles relevant to the topic were examined. No records were unavailable for retrieving. Finally, 109 were included in the review [2,3,4,7,8,9,10,11,12,13,14,15,16,17,18,19,20,21,22,23,24,25,26,27,28,29,30,31,32,33,34,35,36,37,38,39,40,41,42,43,44,45,46,47,48,49,50,51,52,53,54,55,56,57,58,59,60,61,62,63,64,65,66,67,68,69,70,71,72,73,74,75,76,77,78,79,80,81,82,83,84,85,86,87,88,89,90,91,92,93,94,95,96,97,98,99,100,101,102,103,104,105,106,107,108,109,110,111,112]. A detailed flowchart of the search process is shown in Figure 3.

In accordance with the National Institute of Health’s quality assessment tool for Observational Cohorts and Cross-Sectional Studies [6], 20 studies (18.3%) were deemed of Good quality, 69 (63.4%) Fair, and 20 studies (18.3%) as Poor, due to the lack of reporting clinical data (Table S1).

### 3.3. Included Studies’ Characteristics

Among the 110 studies included in the qualitative analysis, 99 were case papers [2,4,7,8,9,10,11,12,13,14,16,17,18,20,21,22,23,24,25,26,27,28,29,30,31,32,33,34,35,36,37,38,39,41,42,43,44,45,46,47,48,49,50,51,52,53,54,56,58,59,60,61,62,63,64,65,66,67,68,69,70,71,72,73,74,75,77,79,80,81,82,83,84,85,86,87,88,89,90,92,93,94,95,96,97,98,99,100,101,103,104,105,107,108,109,110,111,112], while only 11 studies were case series [3,15,19,40,55,57,76,78,91,102,106]. These studies were published between 1949 and 2023 (see Table 1).

### 3.4. Included Patients’ Characteristics

The total number of patients was 184, where 124 (67.4%) were male and 54 female (29.3%). The mean age of the patients was 12.06 ± 3.29 years (range 1.92–18 years) (refer to Table 2). 

The most common etiology was acute rhinosinusitis (69.35%) described in terms of pansinusitis, while frontal acute rhinosinusitis was counted in 22 patients (11.89%). Chronic rhinosinusitis as etiology was clearly stated for six patients (3.26%). Head trauma was found to be the most common cause excluding sinusitis (12; 6.52%). Other etiologies are reported in Table 2. 

### 3.5. Imaging Assessment

In the current analysis, the majority of authors relied on ce-CT as the primary imaging modality for diagnosing PTT and its complications, accounting for 86 cases (46.73%). Additionally, a combination of ce-CT and ce-MRI was utilized in 17.93% of cases (refer to Table 1).

### 3.6. Pathogens

**Figure 4:**
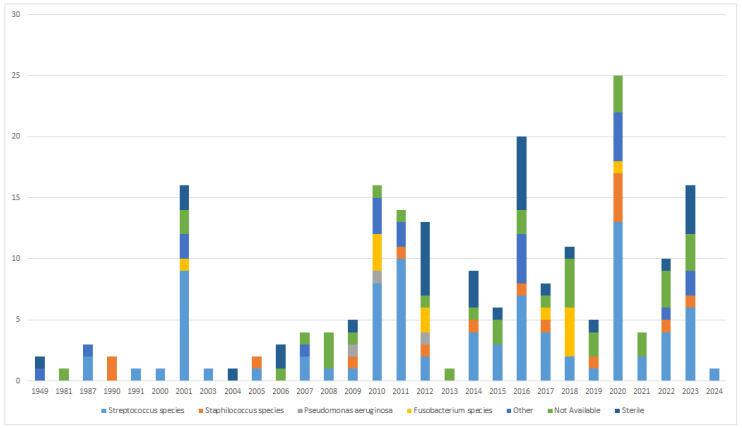
Changes in the various pathogens identified over time.

Microbiological analysis frequently resulted in multiple growth, with streptococci being the most prevalent individual pathogens (85, 40.66%). Among streptococci, *Streptococcus intermedius* was the most frequently cultured (11.96%), while staphylococci accounted for 9.38% of cases. Sterile cultures were prevalent (14.35%) (refer to Table 3). As illustrated in Figure 4, there has been no substantial variation in pathogens over time, with the most frequent being those from the Streptococcus species.

### 3.7. Medical Treatment

All patients received antibiotic therapy, with the duration of treatment ranging from 10 days to 6 months, averaging 6.8 weeks. The predominant antibiotics utilized were ceftriaxone (20.73%) and metronidazole (20.73%), either individually or in combination (see Table 4).

### 3.8. Intracranial Extension

Based on our examination, 131 (71.19%) out of the patients considered in this analysis experienced intracranial complications. Among them, 38 patients were ≤10 years of age (29.00%), 81 patients (61.83%) were between 11 and 18 years old, and 6 patients (4.58%) did not have their age reported.

The predominant intracranial complication observed was epidural abscess (42.59%), succeeded by subdural empyema (17.12%), thrombosis of the superior sagittal sinus (6.48%), and brain abscess (5.55%). Multiple intracranial complications were identified in 38 patients (29.00%). Age, sex, type of imaging assessment, pathogen type, and culture species were not statistically correlated with the development of intracranial complications (*p* value > 0.05). Intracranial complications were significantly associated with the type of surgical treatment (*p* value < 0.0001). Bonferroni correction for multiple comparisons showed the preference for a combined surgical approach in patients with intracranial complications than in those without intracranial involvement, compared to external (*p* = 0.022) and endoscopic interventions (*p* = 0.0002). The details of the intracranial complications from the studies included are provided in Table 5. No other relevant associations were found between intracranial complications and the clinical variables considered (Table 6).

### 3.9. Surgical Treatment

Only seven patients (3.80%) did not undergo any surgery, and out of these, four (2.17%) had intracranial involvement. The type of surgery was not available for 10 cases (5.43%). In all other instances, surgery using various approaches was carried out.

The majority of the authors opted for an external surgical approach for draining subperiosteal abscesses (11.41%). Some authors employed endoscopic endonasal treatment either independently (15.21%) or in conjunction with external drainage (9.78%). Regarding intracranial complications, craniotomy was the primary surgical method in most articles, either on its own (9.23%) or in combination with external drainage (11.41%), endonasal surgery (15.21%), or as a combination of all three modalities (10.32%). Additional combinations of surgeries are detailed in Table 7.

## 4. Discussion

### 4.1. Epidemiology

**Figure 5:**
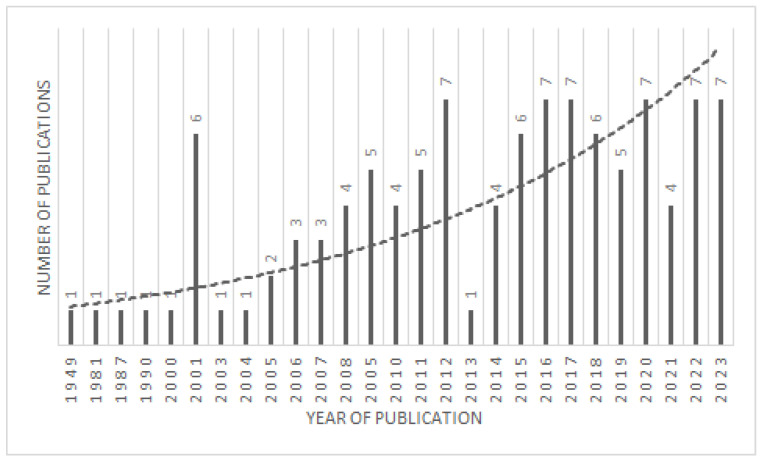
Graph showing the number of published papers trending.

The determination of frequency measures for PPT is challenging due to its rare occurrence. Although it is experiencing minor annual variations, there seems to be an upward trend in the reported cases of PPT in recent years, as illustrated in Figure 5. While these fluctuations may be influenced by publishing pattern, they underscore the significance of promptly recognizing factors that could contribute to PPT predisposition. Frontal sinuses become pneumatized at 6 years of age, and they reach their adult configuration at the age of 15 [28]; that is why teenagers are especially affected by this entity. To our knowledge, only two cases have been reported in children younger than 3 years of age in the literature in the post-antibiotic era [22,50].

### 4.2. Pathophysiology

PPT typically manifests following sinusitis, particularly in cases of pansinusitis. Originating from the frontal sinus, the infection progresses through the frontal bone marrow cavity, inducing osteomyelitis that erodes the external table, leading to the formation of a subperiosteal abscess. Additionally, the infection may extend to the posterior table, giving rise to an epidural abscess. Despite the relative impermeability of the dura mater and arachnoid membranes, the infection can breach these barriers, spreading to the subdural space and causing subdural collections or cerebritis [28].

Consideration should also be given to the hematogenous route, as valveless diploic veins can become infected, resulting in septic thrombophlebitis of the sagittal sinus, subdural empyema, and brain abscess [113]. This phenomenon is more common in children than adults [113]. Persistent bacterial overgrowth in the frontal sinus cavity and adjacent soft tissues allows for small vessel thrombosis and venous congestion [48]. The disruption of the frontal periosteal blood supply initiates an inflammatory response characterized by increased intraosseous pressure, leading to extensive necrosis of the trabecular bone matrix. The resulting avascular and ischemic conditions favor the transition from an aerobic to an anaerobic environment, promoting the growth of opportunistic microorganisms that give rise to abscesses and cortical sinus tracts.

In certain instances, the infection can involve the floor of the frontal sinus, extending to the orbits and causing either orbital cellulitis or an orbital abscess [113].

These theories may partly explain the difference in the incidence of intracranial complications between adult and juvenile populations. In fact, a recent systematic review on intra-orbital complications of Pott’s Puffy Tumor in adults reported an incidence of around 30%, compared to approximately 70% in our review of younger patients [114]. In our study, the proportion of cases in individuals under 18 years of age was 59.5% (184/309), while cases in adults accounted for 40.5% (125/309) [114].

### 4.3. Clinical Presentation 

A gradually tendered tumefaction of the scalp at the forehead is a distinctive indicator of PPT. The initial symptoms and signs of PPT often present subtly, resembling frontal sinusitis. The onset of pronounced symptoms, especially heightened headache and fever, or the manifestation of signs indicating increased intracranial pressure (such as nausea, vomiting, lethargy), periorbital complaints, or a lack of symptom resolution despite antibiotic treatment, necessitate imaging evaluation to detect potential silent intracranial involvement, even in the absence of overt neurological symptoms [113].

### 4.4. Imaging Modalities

Early diagnosis of PPT is crucial for minimizing morbidity and mortality, necessitating a heightened level of suspicion. Diagnosis primarily relies on a comprehensive evaluation of the patient’s history, clinical examination, and imaging studies. When a subperiosteal abscess is suspected, appropriate imaging is essential to confirm the diagnosis and assess potential complications.

The diagnostic workup should involve a ce-CT scan with brain and bony sequences, as it excels in visualizing bone structures and effectively delineates air–bone and air–soft tissue interfaces crucial for sinus surgeons [109]. CT scans can reveal sinusitis, bone erosion, subperiosteal collections, and intracranial extensions, with osteomyelitis indicated by low-attenuated areas of lytic bone destruction. CT is both quick and widely accessible, with pediatric protocols recommended to minimize radiation exposure in children [4].

MRI offers superior soft tissue resolution, making it the gold standard for detecting intracranial complications, dural sinus thrombosis, and bone edema [52]. However, its drawbacks include increased time consumption, the need for anesthesia in younger children, limitations in evaluation bony destruction, and limited availability, even though it can be used to reduce radiation exposure. MRI venography should be added when clinical suspicion of dural or cavernous sinus thrombosis arises [19]. While ultrasound has been proposed for PPT detection in children [85], its diagnostic value remains inadequately investigated in the literature. Additionally, bone Tc-mMP scintigraphy may offer heightened sensitivity compared to CT in early-stage osteomyelitis, though it proves less sensitive in an acute sinusitis setting [115].

Based on the current review, CT emerges as the most effective and commonly used imaging modality for PPT diagnosis. However, in cases with suspected intracranial involvement, MRI is recommended.

### 4.5. Intracranial Manifestations 

The risk of intracranial complications associated with PPT is noteworthy, with reported incidences ranging from 43% to 85% in various studies [1,52,113,116]. The anterior pericranium is particularly susceptible to infection spread due to its rich venous plexus, directly communicating with the diploic veins of the frontal sinus cavity. This anatomical feature enables retrograde flow of septic emboli into the cranial vault, seeding the intracranial space, with or without concurrent erosion of the posterior table of the frontal sinus. PPT can lead to brain abscess, epidural and subdural abscess, superior sagittal sinus thrombosis, pneumocephalus, and meningitis. Less commonly, it can lead to cerebritis and fistula formation [52].

While the frequency of each type of collection varies in published reports, epidural collections are suggested to be the most common focal intracranial manifestation of PPT [1,52,113,116]. Our review found that 92 patients had epidural abscesses, accounting for over 40% of the total cases and more than half of the patients with intracranial complications.

Intracranial complications are often associated with leukocytosis, elevated ESR, and raised CRP levels [34], indicative of the bacterial origin of the complication.

Given the high incidence of seizures in intracranial abscesses (ranging from 19% to 80% of affected patients), immediate initiation of anticonvulsant therapy as prophylaxis against seizures is recommended for all patients with intracranial complications of sinusitis [40,117]. Extracranial complications, such as orbital infections, often co-occur with intracranial disease and shape the clinical presentation, particularly in pediatric patients lacking neurological symptoms. Visible craniofacial manifestations of PPT may serve as early indicators for patients, prompting them to seek medical evaluation sooner.

### 4.6. Microbiology

Due to the comparatively lower oxygen concentration in the frontal sinus, microaerophilic streptococci, including alpha-hemolytic Streptococcus, *Peptostreptococcus*, *Bacteroides* spp., and various anaerobes (such as *Prevotella*, *Porphyromonas*, *Fusobacterium*, and *Peptostreptococcus* spp.), were predominantly cultured from sinogenic sources. Additionally, less-frequently encountered organisms included *Hemophilus influenza*, *Staphylococcus aureus*, and *Enterococcus* spp. [2,15,118].

In our review, the most prevalent bacteria were identified as Streptococcus species, notably *Streptococcus intermedius*, accounting for 11.96% of cases. The prompt initiation of empirical intravenous antibiotic treatment during the initial surgery likely played a significant role in the absence of identifiable pathogens in the subsequent cultures. 

### 4.7. Treatment

The initial treatment of a patient with a PPT is high-dose intravenous antibiotics. The selected antibiotics should possess the capability to cross the blood–brain barrier and provide coverage against both aerobic and anaerobic bacteria [3]. A commonly chosen combination includes a third-generation cephalosporin, metronidazole, and penicillin or vancomycin [see Table 1]. The antibiotic course is typically extended for a minimum of 6–8 weeks postoperatively [2,118]. The successful management of PPT involves a combination of broad-spectrum antibiotic therapy and surgical intervention. Adequate treatment has significantly reduced the mortality rate of sinogenic intracranial complications from 60% to 3.7% [118]. 

Historically, the osteoplastic flap was a conventional surgical access method to the anterior frontal sinus table in PPT. However, with the introduction of endoscopes and powered instrumentation, these methods have been largely replaced by ESS [106]. ESS is superior to classic techniques, providing effective management of the ostio–meatal complex and in opening the frontal recess, which cannot be externally approached [119]. While ESS is often highly effective, select cases with significant pericranial extension may necessitate an external approach with osteoplasties.

The neurosurgical approach to focal intracranial suppuration, particularly subdural empyema, is a subject of debate. Craniotomy is favored in former series examining subdural empyema secondary to sinusitis [117,120]. Small intracranial involvement without focal neurological deficits may initially be managed conservatively but may require craniotomies for abscesses refractory to medical management. Joint neurosurgical drainage and endoscopic or external sinus drainage have been shown to be more effective, leading to faster recovery and shorter hospital length of stay [121].

Frontal lobe abscesses can be treated based on the patient’s condition and the maturity of the abscess wall, either through aspiration with radiological localization or total excision via craniotomy. In our study, we observed that in the presence of intracranial complications, there is a higher prevalence of using a combined approach. This suggests that employing multiple techniques may be more effective for draining intracranial abscesses, whether epidural, subdural, or cerebral. Regardless of the principal treatment, the primary goal remains to drain the abscess and re-establish adequate frontoethmoidal drainage through surgical opening of the sinuses and prolonged antibiotic treatment. Successful treatment of PPT necessitates close collaboration between otorhinolaryngologists, neurosurgeons, pediatricians, bacteriologists, and other related departments.

### 4.8. Limitations of the Study

The present study has several limitations that are worthy of mention:

- Case reports: the review primarily included case reports and short case series, which can limit the generalizability of findings due to variability and heterogeneity.
- Missing data: some older articles lacked details relevant to the study, leading to the exclusion of some information from the analyses.
- Evolution of treatment: the evolution of surgical technique and overall management of PPT that has taken place over the last decades may have improved outcomes, potentially affecting the comparability of older data with more recent data.

## 5. Conclusions

The rarity of PPT poses challenges in defining its frequency accurately, with reported cases displaying an upward trend in recent years. Early diagnosis is imperative for mitigating morbidity and mortality, requiring a heightened level of suspicion. Imaging, particularly contrast-enhanced CT scans, plays a crucial role in confirming the diagnosis and evaluating potential complications.

The risk of intracranial complications in PPT is significant, emphasizing the necessity for a combined approach involving high-dose intravenous antibiotics and surgery. This strategy is crucial in preventing long-term neurological complications and sequelae. Collaborative efforts among multidisciplinary teams, including otorhinolaryngologists, neurosurgeons, pediatricians, and bacteriologists, are indispensable for effective patient management.

## Figures and Tables

**Table 1:** Studies included in the systematic review.

Author	Year	Age (Years)	Sex	Etiology	Imaging	Complication	Surgery	Treatment	Culture
Adnani et al. [7]	2023	8	F	ARS	ce-CT	PSC	NA	NA	NA
Allfather et al. [8]	2017	7	F	FARS	ce-CT	EDA	ESS, CRA	MER, VAN	*Staphylococcus*, *Fusobacterium*
AlMoosa et al. [9]	2016	9	F	T	ce-CT, ce-MRI	None	ESS, CRA	empirical: CLI, CEF; AC: VOR	*Aspergillus Fumigatus*
Amstrup et al. [10]	2023	9	M	ARS	ce-CT, ce-MRI	SSST, EDA	None	CEF, MET	*S. anginosus*
Arnold et al. [11]	2009	10	M	T	CT	EDA	EXD, CRE	CEF, VAN, MET	*S. intermedius*
Arora et al. [12]	2014	14	M	FARS	ce-CT	None	EXD	CEF, CLI	NA
Avcu et al. [13]	2015	12	M	ARS	ce-CT, ce-MRI	PSC	EXD, ESS	CEF, VAN, MET	NA
Bağdatoğlu et al. [14]	2001	18	M	ARS	ce-CT	EDA, SDA	CRA	empirical: IM gentamycin AC: VAN, RIF, MET	STE
Bambakidis et al. [15]	2001	11	M	ARS	CT, only 2 pts MRI	SDA, OA	CRA	BSATB	*Fusobacterium*, *S. pneumoniae*
		11	M	ARS		EDA, SDA	CRA	BSATB	*S. milleri*
		16	M	ARS		SDA	EXD, CRA	BSATB	*Klebsiella* sp., *Peptostreptococcus*
		18	M	ARS		EDA	EXD, CRA	BSATB	*S. microaerophilic*
		15	M	ARS		EDA	CRA	BSATB	*Peptostreptococcus*
		14	M	ARS		EDA	CRA	BSATB	*S. viridans*
		11	F	ARS		EDA, SDA, BA	CRA	BSATB	*S. pyogenes*
Behbahani et al. [3]	2020	7	M	ARS	ce-CT or ce-MRI	EDA	ESS, CRA	CEF	*S. pyogenes*, *Corynebacterium*, *Pseudodiphtheriticum*
		13	M	ARS		SDA	ESS, CRA	CEF, MET	*S. anginosus*
		14	M	ARS		EDA	ESS, CRA	CEF, MET	*S. intermedius*
		6	M	ARS		EDA	ESS, CRA	CEF	*S. pyogenes*
		12	M	ARS		EDA, SDA	ESS, CRA	CEF, CLI	*S. anginosus*
		14	M	ARS		CER	ESS, CRA	CEF, MET	*S. anginosus*, *S. intermedius*, *Staph. epidermidis*
		13	M	ARS		EDA	ESS, CRA	CEF, MET	*S. intermedius*
		10	F	ARS		EDA, CER	ESS, CRA	CEF, MET	*S. intermedius*
		11	M	ARS		EDA	ESS	CEF, MET	*Staph epidermidis*, *Propionibacterium*, *Avidum Diphtheroids*
		5	M	ARS		CER	ESS	CEF, MET	*S. pyogenes*
		5	F	ARS		EDA, MEN	ESS	CEF, MET	*S. intermedius*
		12	M	ARS		SDA	ESS, CRA	CEF, MET	*S, intermedius*, *Staph. aureus*, *Staph. epidermidis*
Belharti et al. [16]	2023	15	M	FARS	ce-CT	EDA	NA	NA	NA
Bhalla et al. [17]	2016	5	M	ARS	ce-CT	subperiosteal OA, EDA	ESS	CEF, CLI, MET	NA
Blackman et al. [18]	2005	9	M	ARS	ce-CT	OC	EXD	CEF, VAN, MET	*Staph. saccharolyticus*
Blumfield et al. [19]	2011	9–16	8M 1F	ARS	8 ce-CT, 1 ce-MRI	2 SSST 6 EDA 1 SDA	3: EXD, ESS, CRA 2: ESS, 2: EXD, CRE 1: none, 1: transferred to another hospital	BSATB	*S. milleri*, *S. pneumonia*, *S. group F*, *Staph. aureus*
Butskiy et al. [20]	2017	10	M	ARS	ce-CT	None	EXD	CEF, CLI	*S. intermedius*
Cannon et al. [21]	2017	5	M	ARS	ce-MRI	None	EXD	NA	*S. anginosus*
Cheng et al. [22]	2009	32 months	M	SEP	ce-CT	SSST	EXD	CLI	*Staph. aureus*
Costa et al. [23]	2020	13	M	ARS	ce-CT	BA, OA	EXD, ESS, CRA	CEF, VAN, MET	NA
Davidson et al. [24]	2006	14	F	ARS	ce-CT	EDA, fistula	CRE	NA	NA
Dayan et al. [25]	2020	4	F	ARS	ce-CT	None	EXD	BSATB	NA
Durur-Subasi et al. [26]	2008	14	F	ARS	ce-CT	EDA, BA	EXD	NA	NA
Faridi et al. [27]	2022	4	F	ARS	ce-CT, ce-MRI	CER	EXD	CEF, VAN	NA
Feder et al. [28]	1987	6	F	ARS	X-ray, ce-CT	None	ESS	empirical: cefuroxime and gentamicin; AC: CLI	*Peptostreptococcus*, *Bacteroides melaninogenicus*
		12	F	ARS	ce-CT	EDA	ESS, CRA	empirical: CLI AC: penicillin	*Alpha Hemolytic S.*
Forgie et al. [29]	2008	16	M	ARS	NA	None	EXD	BSATB	NA
Fu B. [30]	2010	16	M	FARS	ce-CT	None	EXD	BSATB	NA
Fullerton et al. [31]	2016	11	M	FARS	ce-MRI	EDA	EXD, ESS, CRA	CEF	*S. pyogenes*
Gildener-Leapman et al. [32]	2012	5	M	FARS	ce-CT	EDA, SDA, CER	EXD, ESS, CRA	CEF, VAN, MET	*S. intermedius*
Gozgec et al. [33]	2022	15	M	ARS	ce-CT	OC	EXD	BSATB	NA
Guillén et al. [34]	2001	12	F	ARS	ce-CT	OC, EDA, SDA	CRA	CEF, VAN, MET	NA
Gupta et al. [35]	2004	3	M	FARS	ce-CT, ce-MRI	EDA	ESS, CRA	CEF, MET, cloxacillin	STE
Haider et al. [36]	2012	14	M	ARS	ce-CT, ce-MRI	MEN, SDA	EXD, ESS, CRA	MER, MET	*F. necrophorum*
Hassan et al. [37]	2020	15	M	T	ce-CT, ce-MRI	EDA	EXD, ESS	empirical: VAN, AMPS AC: CEF, MET	*F. nucleatum*
Hayek et al. [38]	2007	9	M	ARS	ce-CT	PSC	EXD, ESS	AMPS, then CEF	NA
Heale et al. [39]	2015	5	F	NA	ce-CT	PSC, EDA	EXD, CRA	CEF, ET	*S. anginosus*
Hicks et al. [40]	2011	NA	NA	ARS	ce-CT, MRI	EDA, OC	ESS	BSATB	*S. milleri*
		NA	NA	ARS	ce-CT, MRI	EDA. SDA, SSST	EXD, ESS	BSATB	*S. milleri*
		NA	NA	ARS	ce-CT	SDA	ESS, CRA	BSATB	*S. milleri*
		NA	NA	ARS	ce-CT, MRI	EDA, SDA	ESS, CRA	BSATB	*Cutibacterium acnes*
		NA	NA	ARS	ce-CT, MRI	SDA	EXD, ESS	BSATB	NA
		NA	NA	ARS	ce-CT	BA	CRA	BSATB	*S. milleri*
Hitti et al. [41]	2010	6	F	ARS	ce-CT	EDA	ESS, CRE	empirical: CEF, VAN, MET AC: AMPS	*S. pyogenes*
Holder et al. [42]	1991	17	M	ARS	X-ray, ce-CT	BA	ESS	empirical: ceftazidime and MET AC: ceftazidime, MET, penicillin, chloramphenicol	*Non-hemolytic S.*
Hore et al. [43]	2000	12	F	ARS	CT	None	EXD, ESS	CEF, MET	*S. milleri*
Huijssoon et al. [44]	2003	8	M	ARS	ce-CT	OC	EXD	BSATB	*S. milleri*
Ikoma et al. [45]	2020	12	M	FARS	ce-CT, ce-MRI	EDA, PNC	EXD, CRA	MER, VAN then CEF, MET	*S. constellatus*
Is et al. [46]	2007	11	F	NA	ce-CT	EDA	EXD	AMP, CEF, MET	*Peptostreptococcus*, *Veillonella*, *E. Coli*
Jafri et al. [47]	2015	11	F	ARS	ce-CT	EDA	EXD, CRA	AMO	*S. intermedius*
Joo et al. [48]	2019	7	F	FARS	ce-CT	OC	EXD	AMPS	NA
Kalkan et al. [49]	2017	14	M	ARS	ce-CT, ce-MRI	EDA	ESS, CRA	BSATB	STE
Karadaghy et al. [50]	2022	23 months	M	ARS	ce-CT	PSC	EXD, ESS	AMPS, then LEV	*S. intermedius*, *Granulicatella adiacens*
Karaman et al. [51]	2008	7	F	ARS	ce-CT	None	EXD, ESS	AMPS	*S. milleri*
Ketenci et al. [52]	2011	12	F	ARS	ce-CT	SDA, BA	EXD, ESS, CRA	AMPS, VAN, MET	*Peptostreptococcus*
		13	M	ARS	ce-CT	SDA, BA	EXD, ESS, CRA	CEF, AMPS, MET	*S. pyogenes*
Khan et al. [53]	2006	10	F	MAS	ce-CT	EDA	EXD, CRA, Cortical Mastoidectomy	CEF, CLI	STE
Kim et al. [54]	2012	18	M	ARS	ce-MRI	EDA, SDA	EXD, CRA	AMO, then VAN, ceftazidime, MET	STE
Klivitsky et al. [55]	2023	11	M	ARS	ce-CT, ce-MRI	PSC, SSST, CST	EXT, CRA	CEF and MET	*S. pneumoniae*
		9	F	ARS	CT, MRI	None	EXD	CEF and CLI	Negative
		12	F	ARS	ce-CT, MRI	EDA, SSST	EXD, ESS, CRA	CEF and MET	*S. constellatus*
		14	M	ARS	CT, ce-CT, MRI	EDA, BA	ESS	CEF and MET	*S. intermedius*
		15	M	ARS	CT, ce-CT, MRI	EDA	EXD, ESS, CRA	CEF and MET	STE
		13	M	ARS	CT, ce-CT, MRI	EDA	ESS	CEF and MET	*Prevotella*
		17	F	ARS	CT	None	ESS	CEF and CLI	*S. pneumoniae*
		14	M	ARS	CT, MRI	EDA	EXD, ESS, CRA	CEF and MET	STE
		10	F	ARS	CT	EDA	EXD	CEF and MET	STE
		9	M	ARS	CT	PSC	EXXD	cefuroxime and MET	*Staph. aureus*
Kombogiorgas et al. [2]	2006	11	M	ARS	ce-MRI	EDA	EXD, ESS, CRA	CEF, MET	STE
Kuhar et al. [56]	2023	3	F	ARS	ce-CT, ce-MRI	OC, EDA, SSST	EXD, ESS, CRA	CEF	*S. intermedius*
Kühn et al. [57]	2022	6	M	FARS	ce-CT, ce-MRI	SDA, MEN, CER	ESS, CRA	cefotaxime, CLI	*S. intermedius*
		17	M	ARS	ce-CT, ce-MRI	EDA	ESS	CEF	NA
		9	F	ARS	ce-CT, ce-MRI	EDA	ESS, CRA	cefotaxime, CLI	*S. intermedius*
Lang et al. [58]	2001	14	F	FARS	ce-CT	SDA, SSST	EXD, CRA	cephalosporin, MET	STE
		15	F	ARS	ce-CT	SDA, SSST	EXD, CRA	cephalosporin, MET	*S. pyogenes*
		12	F	ARS	ce-CT	EDA, SDA	EXD, ESS, CRA	cephalosporin, MET	*H. influenzae*
Lauria et al. [59]	2014	14	M	FARS	ce-CT	None	EXD, ESS	VAN, MET, ceftazidime, then AMPS	*S. constellatus*
Ling et al. [60]	2021	9	M	ARS	ce-RM	EDA	NA	BSATB	NA
Linton et al. [61]	2019	16	M	T	ce-CT	OA	EXD, ESS	AMOC MET	STE
Liu et al. [62]	2015	10	F	ARS	ce-CT	EDA	ESS, CRA	MER, then cephalexin	*Alpha Hemolytic S.*
Maheshwar et al. [63]	2001	14	M	T	ce-CT	None	ESS	AMOC, then flucloxacillin, fusidic acid, MET, RIF	*S. intermedius*
Marzuillo et al. [64]	2017	9	F	ARS	ce-CT	Fistula	NA	AMOC	NA
McGee et al. [65]	2022	NA	M	ARS	ce-CT	None	EXD, ESS	CEF, MET	*Staph. aureus*
Morley et al. [66]	2009	7	M	ARS	ce-CT, ce-MRI	EDA	CRA	benzylpenicillin, flucloxacillin then CLI	NA
Moser et al. [67]	2009	14	F	ARS	ce-CT	EDA	EXD, ESS	CEF	STE
Moses et al. [68]	2018	15	M	FB	ce-CT	SDA	ESS, CRA	empirical: TAZO AC: MER, MET	*F. necrophorum*
Nastovska et al. [69]	2017	15	M	FARS	ce-MRI	None	ESS, CRA	benzylpenicillin	*S. anginosus*
Nicoli et al. [70]	2014	13	M	FARS	ce-CT, ce-MRI	EDA	ESS, CRA	BSATB	*S. intermedius*
Nourkami-Tutdibi et al. [71]	2020	6	M	FARS	ce-CT, ce-MRI	EDA	CRA	sultamicillin, then cefotaxime, CLI	*S. intermedius*
Olmaz et al. [72]	2019	12	M	ARS	CT- ce-MRI	EDA	CRA	CEF, VAN, MET	NA
Onesimo et al. [73]	2011	8	F	ARS	ce-MRI	EDA	NA	NA	*S. intermedius*
Özkaya Parlakay et al. [74]	2012	13	M	ARS	ce-CT	None	None	cefotaxime and VAN	NA
Öztürk et al. [75]	2020	15	M	FARS	ce-CT	None	NA	CEF and teicoplanin	NA
Palabiyik et al. [76]	2016	18	M	ARS	ce-CT, ce-MRI	PSC	ESS	AMPS, MET, or CEF	1 *E. Coli*, 1 *S. Epidermidis*, 6 STE
		17	M	ARS	ce-CT, ce-MRI	None	ESS		
		9	M	ARS	ce-CT, ce-MRI	EDA	EXD, CRA		
		11	F	ARS	ce-CT, ce-MRI	EDA	ESS, CRA		
		7	M	T	ce-CT, ce-MRI	None	ESS		
		17	M	ARS	ce-CT, ce-MRI	None	EXD		
Palacios-García et al. [77]	2019	15	M	ARS	ce-CT	None	EXD, ESS	empirical: CLI AC: LEV	*S. intermedius*
Parida et al. [78]	2012	10	M	ARS	ce-CT	PSC	EXD, ESS	BSATB	*Staph. aureus*
		15	F	CRS	ce-CT	None	EXD, ESS	BSATB	STE
		9	F	ARS	ce-CT	None	ESS	BSATB	STE
		11	M	T	ce-CT	None	EXD	BSATB	*Pseudomonas aeruginosa*
		13	M	ARS	ce-CT	None	ESS	BSATB	STE
Patel et al. [79]	2021	13	M	SD	ce-CT	MEN	ESS	CEF, VAN, MET	NA
Patel et al. [80]	2011	11	M	ARS	ce-CT, ce-MRI	fistula, MEN	EXD, CRE	empirical: AMOC AC: MER, amphotericin B, then fluconazole	*Candida parapsilosis*
Pender [81]	1990	13	M	ARS	CT	None	ESS	oxacillin and ampicillin	*Staph. aureus*, *S-viridans*
		17	M	I	CT	EDA and OA	ESS	cefuroxime and MET	*Coagulase neg Staph.*
Podolsky-Gondim et al. [82]	2018	14	M	ARS	ce-CT, ce-MRI	EDA	EXD, CRA	CEF, oxacillin, MET	Peptostreptococcus
Przybysz et al. [83]	2018	8	F	ARS	CT	None	NA	NA	NA
Queen et al. [84]	2001	14	M	ARS	CT, MRI	SDA	EXD, ESS, CRE	BSATB	NA
Reddan et al. [85]	2018	6	M	ARS	CT	None	ESS	NA	NA
Rogers [86]	1949	11	M	NA	NA	NA	EXD, CRE	NA	*B. Alkaligenes faecalis*
		16	M	NA	NA	NA	EXD, CRE	NA	STE
Rogo et al. [87]	2013	5	F	ARS	CT	EDA	None	MER and VAN	NA
Russ et al. [88]	2022	11	M	I	ce-CT	EDA	ESS, CRA	CEF, VAN, MET	*S. anginosus*
Sabatiello et al. [89]	2010	15	M	ARS	ce-CT	SDA, BA	NA	cefotaxime, CLI	*Peptostreptococcus*, *Fusobacterium*
Sade et al. [90]	2016	12	M	FARS	ce-MRI	BA	NA	NA	NA
Salomão et al. [91]	2014	11	F	CRS	ce-CT	EDA	EXD, CRA	BSATB	STE
		9	M	CRS	ce-CT	EDA	None	BSATB	*S. aureus*
		14	M	FARS	ce-CT	EDA, SDA	EXD, CRA	BSATB	*S. pyogenes*
		12	M	T	ce-CT	EDA	EXD, CRA	BSATB	*S. pyogenes*
		12	M	CRS	ce-CT	EDA	EXD, CRA	BSATB	STE
		13	M	T	ce-CT	EDA, fistula	EXD, CRA	BSATB	STE
Sharma et al. [4]	2017	8	F	FARS	CT, ce-MRI	EDA	ESS, CRA	BSATB	*S. intermedius*
Shehu et al. [92]	2008	10	F	ARS	CT	SDA	EXD, CRA	BSATB	NA
Shemesh et al. [93]	2015	11	M	NA	ce-CT	None	NA	NA	NA
Sheth et al. [94]	2018	15	M	FB	ce-CT	SDA	ESS, CRA	empirical: CEF, VAN, TAZO AC: MER, MET	*F. necrophorum*
		17	M	FARS	ce-CT, ce-MRI	SDA, SSST	CRA	CEF, CLI, VAN	*F. necrophorum*
		14	M	ARS	ce-CT	EDA, PNC, CER	CRA	CEF, VAN, MET	*F. necrophorum*, *S. constellatum*
Silva et al. [95]	2022	16	F	ARS	CT, ce-MRI	SDA, BA	CRA	CEF, MET	None
Stark et al. [96]	2016	14	M	FARS	ce-CT	None	EXD, ESS	empirical: flucloxacillin AC: CLI, RIF	*Staph. aureus*
Stoddard et al. [97]	2019	13	M	FARS	ce-MRI	EDA	CRA	CEF, VAN, MET	*Staph. aureus*
Strony et al. [98]	2007	4	M	ARS	ce-CT	EDA	EXD, CRA	CEF, VAN, MET	*S. viridans*
Sugiyama et al. [99]	2016	17	M	ARS	ce-CT, ce-MRI	EDA, PNC	EXD, ESS	CEF, MET	*Peptostreptococcus*, *Collinsella aerofaciens*, *Staph. lugdunensis*
Suwan et al. [100]	2012	8	F	ARS	CT	EDA	EXD, ESS, CRE	VAN, cefotaxime, MET, then AMPS	*S. constellatus*, *F. necrophorum*
Tibesar et al. [101]	2021	15	M	ARS	ce-CT	PSC	EXD, ESS	CEF, VAN, MET	*S. intermedius*
Tsai et al. [102]	2010	14	M	ARS	ce-CT	SSST	EXD, ESS, CRA	BSATB	*F. nucleatum*
		13	M	T	ce-CT	SDA, BA	CRA	BSATB	*Veillonella* sp., *Peptostreptococcus micros*, *F. nucleatum*, *S. viridans*, *Eikenella corrodens*
		15	M	CRS	ce-CT	SDA	EXD, ESS, CRA	BSATB	*Peptostreptococcus micros*; *Coagulase neg Staph.*
		12	M	CRS	ce-CT	SDA	ESS, CRA	BSATB	*Coagulase neg Staph*., *Prevotella* sp.
		9	F	AP	ce-CT	SDA	CRE, fistula repair	BSATB	*P*. *aeruginosa*
		13	M	ARS	ce-CT	EDA, PNC, SDA	EXD, ESS, CRA	CEF, VAN, then penicillin G	*S. constellatus*, *Beta-hemolytic non-group A streptococci*
Tudor et al. [103]	1981	16	M	T	CT	EDA	EXD, CRA	nafcillin, gentamicin, then AMO	NA
Urík et al. [104]	2015	6	M	ARS	ce-CT	EDA, MEN	EXD	CEF, CLI, oral ketoconazole	STE
Vadiee et al. [105]	2023	12	F	IB	CT, ce-MRI	EDA	CRA	ceftazidime, VAN, MET	*Polybacterial*
van der Poel et al. [106]	2016	7	F	T	ce-CT, ce-MRI	SDA, SSST	ESS, CRE	penicillin	*S. intermedius*
		10	F	ARS	ce-CT, ce-MRI	None	ESS	AMOC	*Commensale flora*
		12	F	ARS	ce-CT, ce-MRI	EDA	ESS, CRE	AMOC, then penicillin, MET	*S. constellatus*
		13	M	ARS	ce-CT, ce-MRI	None	None	AMOC	*Streptococcus*
		17	M	ARS	ce-CT, ce-MRI	EDA	ESS, CRE	penicillin, MET	*S. intermedius*
Vanderveken et al. [107]	2012	5	M	ARS	ce-CT	EDA	EXD, CRA	AMOC	STE
Vaphiades et al. [108]	2023	10	M	ARS	ce-MRI	EDA, SSST, MEN	EXD, CRA	BSATB	NA
Verma et al. [109]	2021	14	M	ARS	ce-CT	EDA	EXD, ESS, CRA	CEF	*Streptococcus*
Verma et al. [110]	2018	15	M	ARS	ce-CT	None	ESS	CEF, VAN, MET	NA
		12	M	AFRS	CT	EDA	ESS	itraconazole	NA
		12	F	ARS	CT	None	ESS	BSATB	STE
Weinberg et al. [111]	2005	11	F	ARS	ce-CT	None	ESS	cefotaxime, VAN, MET	*Group C Beta Streptococcus*
Wu et al. [112]	2009	12	F	AP	ce-CT	None	EXD	ceftazidime and gentamicin	*P. aeruginosa*
Our case	2024	12	M	ARS	ce-CT, ce-MRI	EDA	ESS, CRA	ceftazidime, VAN, MET	*S. intermedius*

Abbreviations: AC = after culture; AFRS = allergic fungal rhinosinusitis; AMO = amoxicillin; AMOC = amoxicillin/clavulanate; AMP = ampicillin; AMPS = ampicillin/sulbactam; AP = acupuncture; ARS = acute pansinusitis; BA = brain abscess; BSATB = IV broad-spectrum antibiotics; ce-CT = contrast-enhanced computed tomography; ce-MRI = contrast-enhanced magnetic resonance imaging; CEF = ceftriaxon; CER = cerebritis; CLI = clindamycin; CRA = craniotomy; CRE = craniectomy; CRS = chronic rhinosinusitis; CST = cavernous sinus thrombosis; EDA = epidural abscess; ESS = Endoscopic Sinus Surgery; EXD = external drainage; F = female; FARS = frontal acute rhinosinusitis; FB = foreign body; FR = full recovery; I = influenza; IB = insect bite; LEV = levofloxacin; M = male; MAS = mastoiditis; MEN = meningitis; MER = meropenem; MET = metronidazole; NA = not available; OA = orbital abscess; OC = orbital cellulitis; PNC = pneumocephalus; PSC = preseptal cellulitis; RIF = rifampicin; S = streptococcus; SEP = septicemia; SD = scuba diving; SDA = subdural abscess; SSST = superior sagittal sinus thrombosis; Staph = staphylococcus; STE = sterile; T = trauma; TAZO = piperacillin/tazobactam; VAN = vancomycin; VOR = voriconazole.

**Table 2:** Demographic data and etiologies of studies included in the systematic review.

Demographic Data	N (Range)	%
Patients	184	
Sex	124M/54F/6NA	
Age	12.06 ± 3.29 (1.92–18)	
Year of publication	1949–2023	
Etiology		
Acute rhinosinusitis	128	69.56
Frontal acute rhinosinusitis	22	11.95
Chronic rhinosinusitis	6	3.27
Trauma	12	6.52
Allergic fungal rhinosinusitis	1	0.55
Acupuncture	2	1.08
Insect bite	1	0.55
Influenza	2	1.08
Mastoiditis	1	0.55
Foreign body	2	1.08
Scuba diving	1	0.55
Septicemia	1	0.55
Not available	5	2.71

Abbreviations: M = male; F = female; NA = not available.

**Table 3:** Pathogens cultured from Pott’s Puffy Tumor.

Pathogens Cultured	N	%
*Streptococcus intermedius*	25	11.96
*Streptococcus pyogenes*	10	4.78
*Peptostreptococcus*	10	4.78
*Streptococcus milleri*	9	4.30
*Streptococcus anginosus*	8	3.82
*Streptococcus constellatus*	7	3.35
*Streptococcus viridans*	4	1.91
*Streptococcus pneumoniae*	4	1.91
*Other Streptococcus species*	8	3.82
*Staphylococcus aureus*	10	4.78
*Staphylococcus Epidermidis*	4	1.91
*Coagulase neg Staph.*	3	1.43
*Other Staphylococcus species*	3	1.43
*Pseudomonas aeruginosa*	3	1.43
*Escherichia Coli*	2	0.95
*Fusobacterium necrophorum*	6	2.87
Other Fusobacterium species	6	2.87
Other	21	10.05
Not available	36	17.22
Sterile	30	14.35

**Table 4:** Medical treatment of Pott’s Puffy Tumor.

Medical Treatment	N	%
Ceftriaxone	68	20.73
Metronidazole	68	20.73
Vancomycin	32	9.75
Clindamycin	20	6.09
Ampicillin-sulbactam	12	3.65
Amoxicillin-clavulanate	9	2.74
Meropenem	7	2.13
Cefotaxime	7	2.13
Ceftazidime	6	1.82
Gentamycin	4	1.22
Rifampicin	3	0.91
Flucloxacillin	3	0.91
Cefuroxime	3	0.91
Piperacillin-tazobactam	2	0.61
Levofloxacin	2	0.61
Benzylpenicillin	2	0.61
Oxacillin	2	0.61
Amoxicillin	2	0.61
Voriconazole	1	0.30
Ampicillin	1	0.30
Beta-lattamic ndd	7	2.13
Cephalosporin ndd	4	1.22
Other	8	2.44
IV broad-spectrum antibiotics	43	13.11
Not available	12	3.65

**Table 5:** Complications associated with Pott’s Puffy Tumor.

Intracranial Complications	N	%
Epidural abscess	92	42.59
Subdural abscess	37	17.12
Brain abscess	12	5.55
Superior sagittal sinus thrombosis	14	6.48
Pneumocephalus	3	1.39
Fistula	4	1.85
Cerebritis	7	3.24
Meningitis	7	3.24
Cavernous sinus thrombosis	1	0.46
Not available	2	0.92
None	37	17.13

**Table 6:** Correlation of demographics and clinical details findings between PPT patients with and without intracranial complications.

		Intracranial Complications (131 Cases)	No Intracranial Complications (53 Cases)	*p* Value
Age	Median (IQR)	12.00 (9–14)	12.00 (10–14)	0.865
Sex	Male	34 (66.7%)	88 (70.4%)	0.626
	Female	17 (33.3%)	37 (29.6%)	
Imaging	TC	39 (78.0%)	64 (57.1%)	0.039
	MRI	2 (4.0%)	9 (8.0%)	
	TC + MRI	9 (18.0%)	39 (34.8%)	
Surgery type	External	15 (34.1%)	25 (20.7%)	<0.0001
	Endoscopic	14 (31.8%)	12 (9.9%)	
	Combined	15 (34.1%)	84 (69.4%)	
Pathogen	Gram+	20 (76.9%)	69 (82.1%)	0.569
	Gram-	2 (7.7%)	8 (9.5%)	
	Other	4 (15.4%)	7 (8.3%)	
Culture	Single pathogen	21 (72.4%)	66 (70.2%)	0.820
	Multiple pathogens	8 (27.6%)	28 (29.8%)	

Abbreviation: IQR = interquartile range.

**Table 7:** Surgical treatment of Pott’s Puffy Tumor.

Type of Surgery	N	%
External drainage	21	11.41
Endoscopic Sinus Surgery	28	15.21
Craniotomy	17	9.23
External drainage + Endoscopic Sinus Surgery	18	9.78
External drainage + craniotomy	21	11.41
Endoscopic Sinus Surgery + craniotomy	28	15.21
External drainage + Endoscopic Sinus Surgery + craniotomy	19	10.32
Craniectomy	2	1.08
External drainage, craniectomy	6	3.26
Endoscopic Sinus Surgery, craniectomy	4	2.17
External drainage + Endoscopic Sinus Surgery + craniectomy	2	1.08
External drainage, craniotomy, Cortical Mastoidectomy	1	0.54
Transferred to other hospital	1	0.54
None	6	3.26
Not available	10	5.43

## Data Availability

Data are available at request.

## Supplementary Materials

The following supporting information can be downloaded at: https://www.mdpi.com/article/10.3390/jcm13216428/s1, Table S1: Quality Assessment.
