# Supplementary material for: Pott’s Puffy Tumor in Young Age: A Systematic Review and Our Experience
Source: J Clin Med. 2024 Oct 26;13(21):6428. doi: 10.3390/jcm13216428 (PMC11546441; doi:10.3390/jcm13216428)
Supplement: Supplementary file 1 [file jcm-13-06428-s001.zip › jcm-3281159-supplementary.pdf]

SUPPLEMENTARY MATERIAL

Assessment of quality of included studies according to the National Institutes of Health quality assessment tool for Observational Cohorts and Cross-Sectional Studies [6].

Author	Year	Quality
Adnani et al. [7]	2023	Poor
Allfather et al [8]	2017	Fair
AlMoosa et al. [9]	2016	Good
Amstrup et al. [10]	2023	Fair
Arnold et al. [11]	2009	Fair
Arora et al. [12]	2014	Fair
Avcu et al. [13]	2015	Good
Bağdatoğlu et al. [14]	2001	Fair
Bambakidis et al. [15]	2001	Good
Behbahani et al. [3]	2020	Good
Belharti et al. [16]	2023	Poor
Bhalla et al. [17]	2016	Fair
Blackman et al. [18]	2005	Fair
Blumfield et al. [19]	2011	Good
Butskiy et al. [20]	2017	Fair
Cannon et al. [21]	2017	Poor
Cheng et al. [22]	2009	Fair
Costa et al. [23]	2020	Fair
Davidson et al. [24]	2006	Poor
Dayan et al. [25]	2020	Poor
Durur-Subasi et al. [26]	2008	Poor
Faridi et al. [27]	2022	Fair
Feder et al. [28]	1987	Fair
Forgie et al. [29]	2008	Poor
Fu B. [30]	2010	Poor
Fullerton et al. [31]	2016	Fair
Gildener-Leapman et al. [32]	2012	Good
Gozgec et al. [33]	2022	Fair
Guillén et al. [34]	2001	Fair
Gupta et al. [35]	2004	Fair
Haider et al. [36]	2012	Good
Hassan et al. [37]	2020	Fair
Hayek et al. [38]	2007	Fair
Heale et al. [39]	2015	Fair
Hicks et al. [40]	2011	Good
Hitti et al. [41]	2010	Fair
Holder et al. [42]	1991	Fair
Hore et al. [43]	2000	Fair
Huijssoon et al. [44]	2003	Fair
Ikoma et al. [45]	2020	Fair
Is et al. [46]	2007	Good

Jafri et al. [47]	2015	Fair
Joo et al. [48]	2019	Fair
Kalkan et al. [49]	2017	Fair
Karadaghy et al. [50]	2022	Good
Karaman et al. [51]	2008	Fair
Ketenci et al. [52]	2011	Good
Khan et al. [53]	2006	Good
Kim et al. [54]	2012	Fair
Klivitsky et al. [55]	2023	Fair
Kombogiorgas et al. [2]	2006	Fair
Kuhar et al. [56]	2023	Fair
Kühn et al. [57]	2022	Good
Lang et al. [58]	2001	Fair
Lauria et al. [59]	2014	Fair
Ling et al. [60]	2021	Poor
Linton et al. [61]	2019	Fair
Liu et al. [62]	2015	Fair
Maheshwar et al. [63]	2001	Fair
Marzuillo et al. [64]	2017	Poor
McGee et al. [65]	2022	Fair
Morley et al. [66]	2009	Fair
Moser et al. [67]	2009	Fair
Moses et al. [68]	2018	Good
Nastovska et al. [69]	2017	Poor
Nicoli et al. [70]	2014	Fair
Nourkami-Tutdibi et al. [71]	2020	Fair
Olmaz et al. [72]	2019	Fair
Onesimo et al. [73]	2011	Poor
Özkaya Parlakay et al. [74]	2012	Fair
Öztürk et al. [75]	2020	Poor
Palabiyik et al. [76]	2016	Fair
Palacios-García et al. [77]	2019	Fair
Parida et al. [78]	2012	Fair
Patel et al. [79]	2021	Fair
Patel et al. [80]	2011	Fair
Pender [81]	1990	Fair
Podolsky-Gondim et al. [82]	2018	Good
Przybysz et al. [83]	2018	Poor
Queen et al. [84]	2001	Poor
Reddan et al. [85]	2018	Poor
Rogers [86]	1949	Poor
Rogo et al. [87]	2013	Fair
Russ et al. [88]	2022	Fair
Sabatiello et al. [89]	2010	Fair
Sade et al. [90]	2016	Poor
Salomão et al. [91]	2014	Good

Sharma et al. [4]	2017	Fair
Shehu et al. [92]	2008	Poor
Shemesh et al. [93]	2015	Poor
Sheth et al. [94]	2018	Fair
Silva et al. [95]	2022	Good
Stark et al. [96]	2016	Fair
Stoddard et al. [97]	2019	Fair
Strony et al. [98]	2007	Fair
Sugiyama et al. [99]	2016	Fair
Suwan et al. [100]	2012	Fair
Tibesar et al. [101]	2021	Fair
Tsai et al. [102]	2010	Good
Tudor et al. [103]	1981	Fair
Urík et al. [104]	2015	Fair
Vadiee et al. [105]	2023	Fair
van der Poel et al. [106]	2016	Good
Vanderveken et al. [107]	2012	Fair
Vaphiades et al. [108]	2023	Fair
Verma et al. [109]	2021	Fair
Verma et al. [110]	2018	Fair
Weinberg et al. [111]	2005	Good
Wu et al. [112]	2009	Fair
